# W. Solomon, C. Holland, M. J. Middleton: Autism and Understanding: The Waldon Approach to Child Development: SAGE Publications, London, 2012, 240 pp, $50.00 (paper), ISBN-10: 1446209245

**DOI:** 10.1007/s10803-015-2658-4

**Published:** 2015-11-24

**Authors:** Daniel S. Posner

**Affiliations:** 98 Riverside Dr. #1A, New York, NY 10024 USA

In recent years there has been growing evidence that early problems in motor development are not only common but nearly ubiquitous in ASD children and not only correlated with, but predictive of later diagnostic severity (Lord et al. 2013). This may explain the revival of interest in the “movement perspective” in ASD studies (Torres and Donnellan 2013) and the renewed appreciation of the central role of exploratory movement in child development. Few motor-based, cost-effective, and parent-deliverable approaches that presume competence for learners across the spectrum and promote the development of “on-line” or embodied cognition (Klin et al. 2003) currently exist. *Autism and Understanding*, with an insightful foreword by the esteemed developmental psychobiologist Colwyn Trevarthen, whose work has attended to the role of movement in learning and communication, may expand the treatment options available to parents and practitioners alike.

The book documents an extraordinary and virtually unknown chapter in the history of the treatment of developmental disorders beginning in 1970, when a pioneering neurologist by the name of Geoffrey Waldon, left his post as a consultant at the audiology clinic at the University of Manchester and set out to reform the prevailing educational approach to “backward” children. Part memoir, part oral-history and part theoretical exegesis, *Autism and Understanding* describes Waldon’s movement-based educational approach through the lens of its lead author, Walter Solomon, who deftly balances the telling of his own son’s story of gradual emergence from “remoteness” with colorful and detailed interviews of an entire cohort of special educators, mental health workers, researchers, parents and patients who adopted Waldon’s approach, including approximately 30 longitudinal case descriptions of children of varying ages and clinical impairment. At times the narrative, especially the opening memoir section, teeters toward the overfamiliar; the story of anxious parents coming painfully to terms with a diffident and sometimes blaming medical establishment doesn’t feel as newsworthy as it might have even a decade or so ago. The book finds its real center of gravity when we are finally introduced to the arresting figure of Waldon himself in chapter 4, at which point the authors shift stylistic gears and bear down on what feels like the core content of the book—the unique developmental theory and practice of the Waldon Approach. In three brisk but dense chapters that follow, they manage to provide a detailed breakdown of the scientific underpinnings of Waldon’s theory in language accessible enough for parents and rigorous enough to satisfy the informed researcher/practitioner.

The Waldon Approach presumes the primacy of movement in early childhood development. A careful observer of “neuro-typical” as well as “neuro-atypical” children, Waldon noticed that when very young children could not move as intended they would not go on to explore their surroundings with the sort of vigor and wide-ranging curiosity of their typical peers—and would often develop repetitive, avoidant and what Waldon termed “self-delighting” behaviors. Perhaps because of his neurological background, Waldon appreciated that the capacity for self-movement was inextricably linked to the quality of the sensory feedback produced by one’s own movements—a recursive feature of the animal nervous system that Charles Sherrington, working at the turn of the twentieth century famously termed proprioception. Without intact proprioception, or “self-feeling,” skillful, purposeful movement—something that for typically-developing children is both pleasurable and second-nature—can become laborious. And without skillful, purposeful movement the construction of an intact body schema and functioning sense of peri-personal space may be thwarted. Acts of intentional movement and affective social engagement can be disrupted, requiring ‘autistic’ behavioral and cognitive compensations (Trevarthen and Delafield-Butt 2013).

Waldon’s concern was that the intrinsic motivation to explore and make sense of their surroundings is frequently absent or underdeveloped in children with sensory-motor difficulties. So he devised a particular educative framework that became known as the Waldon Lesson, which, unlike most special education instruction then and now, minimized verbal and social demands and foregrounded the cultivation of the sort of movements that neuro-typical children tend to make in their early years. In particular he emphasized the early “precursor” movements such as acquiring and disposing, banging, scraping and what he called the “continuant behaviour” of toddlers. Echoing Harlow (1949) these led on to “the learning how to learn” tools including scribbling, sorting, matching, sequencing, brick-building and coding. Like his contemporary Piaget, Waldon observed that gradual mastery of these increasingly elaborate movements would promote the development of accessible sensory motor schemas (Piaget 1973), and flexible, on-line intelligence, or what Waldon termed, “general understanding.” His innovation was to assume that atypically-developing children could benefit from practicing these movements in a prepared play environment under the watchful, supportive but un-interfering supervision of an adult.

Waldon’s approach was radical for its time. The book describes frequent encounters between Waldonites and school boards dubious of any approach that did not explicitly instruct and/or foreground behavior modification. But the detailed case summaries in *Autism and Understanding*—as well as Jiri Berger’s 1985 clinical trial^1^—strongly suggest that a large cohort of children with a diverse array of learning impediments and behavioral problems have benefited. Even severely affected children showed evidence of increased spatial and social cognition—one parent recalls her profoundly oblivious son gradually acquiring the “understanding” to notice and step around oncoming pedestrians on the sidewalk—and a range of problem behaviors also seemed to fall away with the steady improvement in “exploratory motor competence” (Bornstein et al. 2013). As Berger points out: “The significant increases in attention, effort and concentration and the corresponding decreases in problem behaviors are…in line with the Waldon theory” (*Autism and Understanding*, p.119) which predicts that enhanced “general understanding” should reduce the anxiety and perplexity behind many maladaptive behaviors. This is an intriguing hypothesis but the authors—eager to cover a wide swath of oral-historical ground—could have done more to unpack or support it. For example, there is now a wealth of evidence, particularly from the field of Dynamic Systems Theory that supports the notion of a self-organizing “motivational cascade” connecting motor and cognitive development (Thelen 2005) but the authors make no reference to this or any other competing hypotheses of cognitive development. In this regard, the book—like the history of the Waldon movement itself—retains a certain hermetic quality.

Shortcomings aside, *Autism and Understanding* offers a valuable and engaging description of a milieu where motor-based therapy has been conceptualized as a primary intervention for what was presciently understood as a disorder of sensory-motor intentionality. (Trevarthen and Delafield-Butt 2013). It should provoke further research and discussion into the role of early interventions that accommodate “movement differences” (Donnellan et al. 2013).

## Footnotes

1 Berger’s unpublished study is available in full on the lead author’s website: autismandunderstanding.com.
